# NAC-MYB-based transcriptional regulation of secondary cell wall biosynthesis in land plants

**DOI:** 10.3389/fpls.2015.00288

**Published:** 2015-05-05

**Authors:** Yoshimi Nakano, Masatoshi Yamaguchi, Hitoshi Endo, Nur Ardiyana Rejab, Misato Ohtani

**Affiliations:** ^1^Graduate School of Biological Sciences, Nara Institute of Science and TechnologyIkoma, Japan; ^2^Division of Strategic Research and Development, Graduate School of Science and Engineering, Saitama UniversitySaitama, Japan; ^3^PRESTO (Precursory Research for Embryonic Science and Technology), Japan Science and Technology AgencyKawaguchi, Japan; ^4^Faculty of Science, Institute of Biological Sciences, University of MalayaKuala Lumpur, Malaysia; ^5^Biomass Engineering Program Cooperation Division, RIKEN Center for Sustainable Resource ScienceYokohama, Japan

**Keywords:** land plant evolution, MYB transcription factor, NAC transcription factor, secondary cell wall, network

## Abstract

Plant cells biosynthesize primary cell walls (PCW) in all cells and produce secondary cell walls (SCWs) in specific cell types that conduct water and/or provide mechanical support, such as xylem vessels and fibers. The characteristic mechanical stiffness, chemical recalcitrance, and hydrophobic nature of SCWs result from the organization of SCW-specific biopolymers, i.e., highly ordered cellulose, hemicellulose, and lignin. Synthesis of these SCW-specific biopolymers requires SCW-specific enzymes that are regulated by SCW-specific transcription factors. In this review, we summarize our current knowledge of the transcriptional regulation of SCW formation in plant cells. Advances in research on SCW biosynthesis during the past decade have expanded our understanding of the transcriptional regulation of SCW formation, particularly the functions of the NAC and MYB transcription factors. Focusing on the NAC-MYB-based transcriptional network, we discuss the regulatory systems that evolved in land plants to modify the cell wall to serve as a key component of structures that conduct water and provide mechanical support.

## Introduction

The cell wall, a characteristic feature of plant cells, consists of biopolymers, such as polysaccharides, phenolic compounds, and various proteins, which impart mechanical strength and rigidity. The structure of the cell wall determines the characteristics of plant cells, thus directly affecting organ development and responses to environmental stimuli (Hamant and Traas, 2010; Wolf et al., 2012).

**Figure 1:**
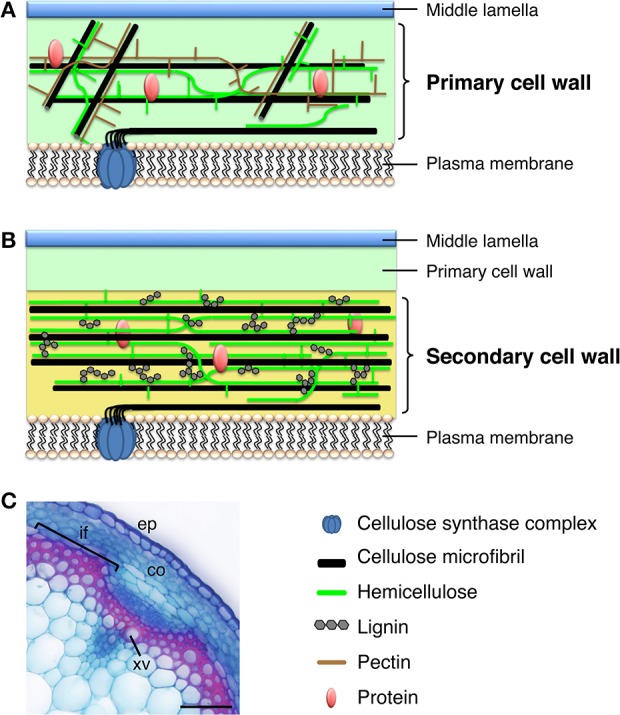
**Plant cell walls. (A)** Model of the primary cell wall. Cellulose microfibrils in the primary cell wall are relatively short and thin, compared with those in the secondary cell wall, and hemicellulose in the primary cell wall is composed of xyloglucan. The primary cell wall is rich in pectin. **(B)** Model of the secondary cell wall, which is deposited between the primary cell wall and the plasma membrane. The secondary cell wall mainly contains relatively long and thick cellulose microfibrils, hemicellulosic xylan, and lignin. **(C)** Cross section of an Arabidopsis inflorescence stem stained with Safranin, which stains lignin red, and Astra blue. co, cortex; ep, epidermis; if, interfascicular fiber; xv, xylem vessel. Bar = 50 μm.

Plant cells have two types of cell wall, primary cell wall (PCW) and secondary cell wall (SCW), based on their biosynthetic composition and cellular location (Figure 1). Every plant cell has a PCW, a relatively thin and extensible wall that the cell synthesizes during cell division. The force generated by the PCW functions as a critical regulator of cell elongation and expansion; thus, PCW biosynthesis fundamentally conditions the shape and size of cells (Geitmann, 2010; Hamant and Traas, 2010). By contrast, the relatively thick and rigid SCW forms in specific types of cells, such as xylem cells, and cells of valve margin and anther endothecium. The cell produces the SCW between the PCW and the plasma membrane during cell differentiation, and the SCW imparts additional mechanical stiffness and/or hydrophobicity to the cell (Cosgrove and Jarvis, 2012). Cellulose, also called (1,4)-β-d-glucan, contains >500 β-d-glucose residues polymerized with glycosidic bonds into a chain; cellulose microfibrils contain ~40 cellulose chains formed into bundles. Cellulose constitutes the main component of the PCW and SCW, but the cellulose of the PCW and SCW shows key structural differences. In the PCW, cellulose has a relatively low degree of polymerization (e.g., 2000–6000 β-d-glucose residues in cotton, *Gossypium hirsutum*) and microfibril widths of 2–2.5 nm. By contrast, in the SCW, cellulose has a high degree of polymerization (e.g., 13,000 β-d-glucose residues in cotton and approximately 8000 in wood; Marx-Figini, 1969) and microfibril widths of 5–10 nm (Heyn, 1955, 1965, 1966). Other components of the cell wall also differ between the PCW and SCW. For example, the PCW typically contains xyloglucan as the major hemicellulose (i.e., the polysaccharide component that is soluble in alkali), but the SCW contains xylan. In addition, the PCW is rich in gel-like pectin, whereas lignin and specific phenolic polymers are more abundant in the SCW (Figure 1). These differences in cell wall composition impart different physical properties to the cell wall, rendering the PCW flexible and the SCW mechanically and biologically robust.

The differences in cell wall components between the SCW and PCW suggest that plants have a set of SCW-specific biosynthetic genes. Indeed, transcriptome analysis of xylem tissues in tree species identified many genes thought to be involved in the biosynthesis of SCW-specific polymers during xylem development in loblolly pine (*Pinus taeda*, Allona et al., 1998; Lorenz and Dean, 2002), poplar (*Populus*, spp., Sterky et al., 2004), white spruce (*Picea glauca*, Pavy et al., 2005), and eucalyptus (*Eucalyptus gunnii*, Rengel et al., 2009). A series of molecular genetic studies on *Arabidopsis thaliana irregular xylem* (*irx*) mutants, in which xylem cells are disrupted due to stunted SCW formation (Turner and Somerville, 1997), expanded our knowledge of SCW-specific enzymes. For example, functional molecular and co-expression analysis of *IRX* genes identified SCW-specific cellulose synthase subunit A (CesA) genes (*IRX1/CesA8*, *IRX3/CesA7*, and *IRX5/CesA4)*, SCW-specific hemicellulose biosynthetic genes (*IRX7*, *IRX8*, *IRX9*, *IRX10*, *IRX14*, and *IRX15*), and lignin biosynthetic genes (*IRX4* and *IRX12*) (Turner and Somerville, 1997; Jones et al., 2001; Brown et al., 2005, 2009, 2011; Lee et al., 2007; Peña et al., 2007; Wu et al., 2009; Jensen et al., 2011). These findings also suggested that the upregulation of SCW-specific enzyme genes promotes SCW formation, and that SCW formation requires this SCW-specific transcriptional regulatory system.

**Figure 2:**
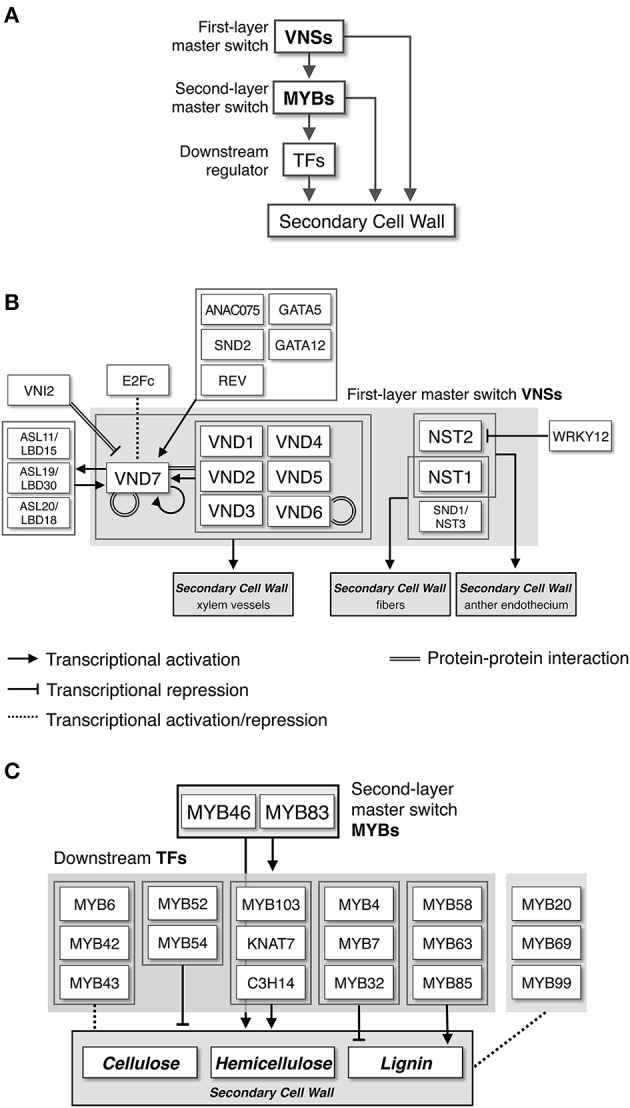
**Transcriptional network regulating secondary cell wall formation. (A)** NAC-MYB-based transcriptional regulation of secondary cell wall biosynthesis. Some metabolic genes for secondary cell wall biosynthesis are targeted by both NACs and MYBs, producing “feed-forward” regulation. **(B)** Transcriptional regulatory network around VNS proteins, first-layer master switches for secondary cell wall formation, based on work in Arabidopsis. **(C)** Transcriptional regulatory network around MYB proteins, second-layer master switches for secondary cell wall formation, based on work in Arabidopsis.

In 2005, a milestone year for SCW biosynthesis research, multiple studies identified transcriptional regulators of SCW biosynthesis. For example, Kubo et al. used an *in vitro* cell culture system to identify the plant-specific NAM, ATAF1,2, and CUC2 (NAC) transcription factors VASCULAR-RELATED NAC-DOMAIN1-7 (VND1-7) as master regulators of xylem vessel cell differentiation (Kubo et al., 2005). Also, Mitsuda et al. reported that NAC SECONDARY WALL THICKENING PROMOTING FACTOR1 (NST1) and NST2, members of a sister group to the VNDs, regulate SCW formation in anther cells (Mitsuda et al., 2005). Subsequent work showed that NST1 and NST3 (also called SECONDARY WALL-ASSOCIATED NAC DOMAIN PROTEIN1 [SND1]) function as master switches of fiber cell differentiation in Arabidopsis (Zhong et al., 2006; Mitsuda et al., 2007). These findings revealed the regulation of SCW biosynthesis at the molecular level, showing that plants have specific transcriptional switches that regulate SCW biosynthesis, and these factors belong to the NAC family, including VND and NST transcription factors (Yamaguchi and Demura, 2010; Zhong et al., 2010a; Wang and Dixon, 2011; Hussey et al., 2013). After these reports on NAC proteins, additional reports implicated several MYB-type transcription factors as secondary master regulators of SCW formation (McCarthy et al., 2009; Ko et al., 2012, 2014; Zhong and Ye, 2012; Hussey et al., 2013), and proposed an intricate network of transcription factors that regulate SCW formation in Arabidopsis (Figure 2).

In this review, we describe the transcriptional regulation of SCW formation based on information accumulated in the decade since 2005, focusing on the well-studied NAC and MYB transcription factors. An analysis of the NAC-MYB-based transcriptional regulatory system of the SCW reveals clues to how plant cells modify cell wall biosynthesis to conduct water (xylem vessels) and/or provide support (fibers).

## The function of NAC (NAM, ATAF1,2 and CUC2) proteins in SCW formation

### VNS (*V*ND, *N*ST/SND, and *S*MB related) proteins function as master regulators of SCW formation

Members of the NAC domain transcription factor family have a highly conserved N-terminal NAC domain, which has been implicated in nuclear localization, DNA binding, and homo- and/or heterodimer formation with other NAC domain proteins (Olsen et al., 2005). NAC transcription factors consist of a large gene family with more than 100 members in *A. thaliana* (Ooka et al., 2003), *Oryza sativa* (Ooka et al., 2003; Nuruzzaman et al., 2010), *Glycine max* (Le et al., 2011), *Populus trichocarpa* (Hu et al., 2010), and *Eucalyptus grandis* (Hussey et al., in press). The NAC proteins have been reported to participate in many developmental processes (Krizek and Fletcher, 2005; Olsen et al., 2005; Petricka et al., 2012), including SCW formation (Yamaguchi and Demura, 2010) and biotic and abiotic stress responses (Fang et al., 2008; Nakashima et al., 2012; Puranik et al., 2012).

**Figure 3:**
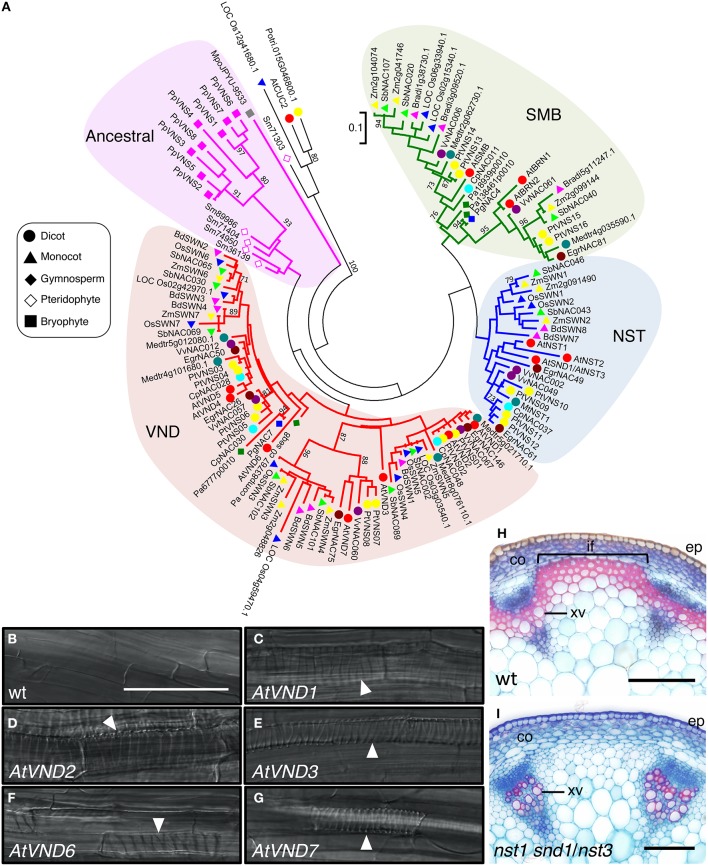
**The *VNS* genes function as first-layer master switches for secondary cell wall formation. (A)** Phylogenetic tree of VNS proteins. The unrooted phylogenetic tree was constructed with amino acid sequences of the NAC domain (sequences provided in Table S1) by the maximum-likelihood method. Numbers indicate bootstrap values for the clades that received support values of over 70% (1000 resamplings). Scale (0.1) represents a 10% change in sequences. Based on the tree, the VNS proteins are classified into four groups, VND, NST/SND, SMB, and Ancestral groups. **(B–G)** Seven-day-old Arabidopsis roots of wild type (wt, **B**) and transgenic plants, in which *AtVND1* **(C)**, *AtVND2* **(D)**, *AtVND3* **(E)**, *AtVND6* **(F)**, and *AtVND7* **(G)** were overexpressed by an inducible system. Ectopic xylem elements formed in the transgenic roots (white arrowheads). Data were adapted from Endo et al. (2015). **(H,I)** Cross sections of Arabidopsis inflorescence stems stained with Safranin, which stains lignin red, and Astra blue. In the wild type (wt), both xylem vessel cells and interfascicular fiber cells have lignin-containing secondary cell wall, thus they stain red **(H)**. By contrast, the mutant *nst1 snd1*/*nst3* lacks secondary cell wall in interfascicular fiber cells, thus only xylem vessel cells were stained by Safranin **(I)**, as described in Mitsuda et al. (2007). co, cortex; ep, epidermis; if, interfascicular fiber; xv, xylem vessel. Bars = 100 μm **(B–I)**.

The first clear indication of NAC protein function in SCW formation came from studies of *in vitro* transdifferentiation of tracheary elements using *Zinnia elegans* mesophyll cells. Demura and co-workers found that expression of the NAC domain transcription factor *Z567* increased during transdifferentiation (Demura et al., 2002). They further established an *in vitro* system for xylem vessel cell differentiation with Arabidopsis suspension culture cells, and showed that expression of seven NAC transcription factors with high sequence similarity to Z567 also increased, beginning in the early stages of cell differentiation (Kubo et al., 2005). They named these proteins VASCULAR-RELATED NAC-DOMAIN1 (VND1) to VND7 (Figure 3A, Table 1). All the *VND* genes are preferentially expressed in developing vascular tissues, although their expression patterns differ; promoter analysis suggested that VND7 regulates all types of xylem vessels in roots and shoots, whereas the other VND proteins might differentially regulate vessel formation (Kubo et al., 2005; Yamaguchi et al., 2008). Overexpression of *VND* genes induces ectopic deposition of patterned SCW, which is characteristic of xylem vessel cells (Kubo et al., 2005; Zhou et al., 2014; Endo et al., 2015; Figures 3B–G). Conversely, overexpression of a dominant chimeric repressor constructed by fusing VND6 or VND7 to the SRDX transcriptional repression domain, severely inhibited xylem vessel cell differentiation (Kubo et al., 2005; Reusche et al., 2012). Together, these findings indicate that the VND proteins act as master regulators of xylem vessel cell differentiation.

**Table 1:** **Known *VNS* genes**.

***VNS* GENES**				**References**
**VND**	**NST**	**SMB**	**Ancestral**	
***Arabidopsis thaliana***				
AtVND1	AtNST1	AtBRN1	–	Kubo et al., 2005
AtVND2	AtNST2	AtBRN2		Mitsuda et al., 2005, 2007
AtVND3	AtNST3/AtSND1	AtSMB		Zhong et al., 2006
AtVND4				Bennett et al., 2010
AtVND5				
AtVND6				
AtVND7				
***Carica papaya***				
CpNAC028	CpNAC037	CpNAC011	–	Zhu et al., 2012
CpNAC030				
CpNAC048				
***Populus trichocarpa***				
PtVNS01/PtrWND5A/PtrVND6-C1	PtVNS09/PtrWND2A/PtrSND1-B1	PtVNS13/PtrSND1-L-1	–	Zhong et al., 2010b
PtVNS02/PtrWND5B/PtrVND6-C2	PtVNS10/PtrWND2BPtrSND1-B2	PtVNS14/PtrSND1-L-2		Ohtani et al., 2011
PtVNS03/PtrWND4A/PtrVND6-B2	PtVNS11/PtrWND1B/PtrSND1-A2	PtVNS15		Li et al., 2012b
PtVNS04/PtrWND4B/PtrVND6-B1	PtVNS12/PtrWND1A/PtrSND1-A1	PtVNS16		
PtVNS05/PtrWND3A/PtrVND6-A1				
PtVNS06/PtrWND3B/PtrVND6-A2				
PtVNS07/PtrWND6A/PtrVND7-2				
PtVNS08/PtrWND6B/PtrVND7-1				
***Medicago truncatula***				
Medtr4g101680.1	MtNST1	Medtr2g062730.1	–	Zhao et al., 2010
Medtr5g012080.1		Medtr4g035590.1		Phytozome v9.1
Medtr5g021710.1				(http://www.phytozome.net/)
Medtr8g076110.1				
***Eucalyptus grandis***				
EgrNAC26	EgrNAC49	EgrNAC81	-	Hussey et al., in press
EgrNAC50	EgrNAC61			
EgrNAC75				
EgrNAC146				
***Vitis vinifera***				
VvNAC012	VvNAC002	VvNAC006	–	Zhu et al., 2012
VvNAC057	VvNAC049	VvNAC061		
VvNAC060				
VvNAC067				
***Zea mays***				
ZmSWN3	ZmSWN1	Zm2g041746	–	Zhong et al., 2011
ZmSWN4	ZmSWN2	Zm2g099144		Zhu et al., 2012
ZmSWN5	Zm2g091490	Zm2g104074		
ZmSWN6				
ZmSWN7				
Zm2g048826				
***Sorghum bicolor***				
SbNAC002	SbNAC043	SbNAC020	–	Zhu et al., 2012
SbNAC030	SbNAC046	SbNAC040		
SbNAC065		SbNAC107		
SbNAC069				
SbNAC089				
SbNAC101				
SbNAC102				
***Oryza sativa***				
OsSWN3	OsSWN1	LOC_Os02g15340.1	–	Zhong et al., 2011
OsSWN4	OsSWN2	LOC_Os06g33940.1		Zhu et al., 2012
OsSWN5				
OsSWN6				
OsSWN7				
LOC_Os02g42970.1				
LOC_Os03g03540.1				
LOC_Os04g59470.1				
***Brachypodium distachyon***				
BdSWN1	BdSWN7	Bradi1g38730.1	–	Valdivia et al., 2013
BdSWN2	BdSWN8	Bradi3g09520.1		Phytozome v9.1
BdSWN3		Bradi5g11247.1		(htt://www.phytozome.net/)
BdSWN4				
BdSWN5				
BdSWN6				
***Picea abies***				
Pa_comp83767_c0_seq8	–	Pa138461p0010	–	Nystedt et al., 2013
Pa6777p0010		Pa18939p0010		
***Picea glauca***				
PgNAC7	–	PgNAC4	–	Duval et al., 2014
***Selaginella moellendorffii***				
–	–	–	Sm36139	Xu et al., 2014
			Sm71404	
			Sm74950	
			Sm89986	
***Physcomitrella patens***				
–	–	–	PpVNS1	Xu et al., 2014
			PpVNS2	
			PpVNS3	
			PpVNS4	
			PpVNS5	
			PpVNS6	
			PpVNS7	
			PpVNS8	
***Marchantia polymorpha***				
–	–	–	MpoJPYU-9533	Xu et al., 2014

In *A. thaliana*, the VND-related proteins NAC SECONDARY WALL THICKENING PROMOTING FACTOR1 (NST1), NST2, and NST3/SECONDARY WALL-ASSOCIATED NAC DOMAIN PROTEIN 1 (SND1)/ARABIDOPSIS NAC DOMAIN CONTAINING PROTEIN012 (ANAC012) (Figure 3A, Table 1) regulate the differentiation of SCW-containing cells other than xylem vessel cells, such as anther endothecium (NST1 and NST2; Mitsuda et al., 2005), fiber cells (NST1 and NST3; Zhong et al., 2006, 2007b; Mitsuda et al., 2007; Figures 3H,I, Table 1), and silique cells (NST1 and NST3; Mitsuda and Ohme-Takagi, 2008). Moreover, other VND-related Arabidopsis proteins, namely SOMBRERO (SMB), BEARSKIN1 (BRN1), and BRN2, induce ectopic SCW deposition when overexpressed, although in wild type cells, they are expressed in root cap regions where SCW is not deposited (Willemsen et al., 2008; Bennett et al., 2010; Figure 3A, Table 1). These results indicate that the capacity to induce SCW biosynthesis is conserved among the VND, NST, SMB, and BRN proteins, and that these genes likely evolved from a common ancestral gene, acquiring the capacity to regulate wall modification during the differentiation of specific cell types.

The NAC protein subfamily, including VND, NST, SMB and BRN of Arabidopsis has been termed the VNS (*V*ND, *N*ST/SND, *S*MB related protein) family (Ohtani et al., 2011; Xu et al., 2014). The same subfamily was called “subfamily Ic” by Zhu et al. (2012), and is conserved among wide range of plant species, including non-vascular land plant species such as Bryophytes (Zhu et al., 2012; Xu et al., 2014; Figure 3A, Table 1).

### VNS proteins are well-conserved among vascular plants

As shown in Table 1, many VNS proteins have been identified in land plant species. The VNS proteins in vascular plants can be classified into VND, NST, or SMB groups by phylogenetic analysis (Figure 3A), suggesting that the diversification of *VNS* genes occurred within the vascular plant lineage.

Black cottonwood (*P. trichocarpa*; *Pt* or *Ptr* in different studies), the first tree species with a high-quality, annotated genomic sequence (Tuskan et al., 2006), contains 16 *PtVNS* genes, including eight genes of the VND group, four genes of the NST group, and four genes of the SMB group (Zhong et al., 2010b; Ohtani et al., 2011; Li et al., 2012b; Figure 3A, Table 1). The *PtVNS* genes include *PtrWND*, *PtrVND6*, *PtrVND7*, and *PtrSND1* and all *PtVNS* genes of the VND and NST groups are expressed in developing xylem and phloem fiber regions, although in primary vessels in the stem, only *PtVNS* genes of the VND group are expressed (Zhong et al., 2010b; Ohtani et al., 2011). The SMB-group *PtVNS* genes are not expressed in the xylem tissues, but rather are expressed in root tissues (Zhong et al., 2010b; Ohtani et al., 2011), indicating that the molecular functions of *VNS* genes in the SMB group in root tissue development might be conserved in Arabidopsis and poplar. Overexpression of *PtVNS* genes of the VND and NST groups in Arabidopsis and poplar caused ectopic deposition of SCW (Zhong et al., 2010b; Ohtani et al., 2011), and *AtNST3* promoter-driven *PtVNS* genes can rescue fiber cell formation in *nst1 snd1/nst3* double mutant stems (Zhong et al., 2010b; Figures 3H,I). Thus, the PtVNS proteins appear to possess the full potential to induce SCW biosynthesis.

Monocot VNS proteins have been also studied in terms of their expression patterns and molecular functions (Zhong et al., 2011; Valdivia et al., 2013; Yoshida et al., 2013). The members of the VND and NST groups in rice, maize (*Zea mays*), and *Brachypodium distachyon* are named SECONDARY WALL-ASSOCIATED NAC (SWN) proteins, and are expressed in SCW-forming cells such as xylem vessels, cortical fibers, and bundle sheath fibers (Zhong et al., 2011; Valdivia et al., 2013; Yoshida et al., 2013). Heterologous overexpression of the *SWN* genes can induce ectopic SCW deposition (Zhong et al., 2011; Valdivia et al., 2013), like the *AtVNS* and *PtVNS* genes; thus the SWN proteins are sufficient to promote the downstream events of SCW formation.

In addition to poplar and monocots, comparative genomics research has identified *VNS* genes in many plant species. The numbers of *VNS* gene vary by plant species without apparent correlation to genome size or the presence of woody tissues (Zhu et al., 2012; Figure 3A, Table 1). Recently, genome sequencing has been completed for other tree species, such as *Picea abies* (Nystedt et al., 2013) and *E. grandis* (Myburg et al., 2014). These tree species have only a few *VNS* genes: four in *P. abies* (Nystedt et al., 2013), six in *E. grandis* (Hussey et al., in press; Myburg et al., 2014), and two in *P. glauca* (Duval et al., 2014). It is noteworthy that no *VNS* genes of the NST group have been identified in gymnosperms at present (Figure 3A); thus, the NST group might have evolved within the angiosperm lineage, or lost in the gymnosperm lineage. The wood of gymnosperms is composed of single cell tracheids that function in water conduction and provide mechanical strength to the axis, whereas the wood of angiosperms is composed of xylem vessels and fibers, cells that are specialized for water conduction and providing mechanical strength, respectively (Pallady, 2008). Phylogenetic analysis suggests that gymnosperm tracheid cell differentiation could be regulated by the VND-type *VNS* genes (Figure 3A). Further studies on gymnosperm *VNS* genes will give insights into how woody cells developed during land plant evolution.

### Target genes of VNS for SCW formation

As mentioned above, the overexpression of *VNS* genes induces ectopic SCW deposition (Kubo et al., 2005; Mitsuda et al., 2005, 2007; Zhong et al., 2006, 2010a,b, 2011; Bennett et al., 2010; Yamaguchi et al., 2010a; Ohtani et al., 2011; Valdivia et al., 2013; Endo et al., 2015; Xu et al., 2014; Figures 3B–G). This observation demonstrates that VNS proteins likely share target genes to produce SCW. Indeed, the overexpression of *AtVND* and *AtNST* commonly upregulates the genes involved in the biosynthesis of SCW components, such as cellulose, hemicellulose and lignin (Kubo et al., 2005; Mitsuda et al., 2005, 2007; Zhong et al., 2006; Ko et al., 2007). However, xylem vessel cells and fiber cells differ in specific cell wall characteristics, such as the syringyl/guaiacyl (S/G) ratio of lignin subunits (Saito et al., 2012). In addition, expression of *AtVND7* under the control of the *AtSND1/NST3* promoter in the *nst1 snd1/nst3* double mutant rescues the lack of SCW in mutant fiber cells (Mitsuda et al., 2007; Zhong et al., 2007b; Yamaguchi et al., 2011; Figures 3H,I). However, the SCW formed in the fibers differs from that produced by expression *AtNST3*; expression of *AtVND7* causes formation of the patterned SCW characteristic of xylem vessel cells, even in the fiber cells (Yamaguchi et al., 2011). Accordingly, parts of the downstream SCW-related pathway appear to differ between AtVND and AtNST.

Around 2010, several groups independently identified the direct target genes of AtVND6, AtVND7, and AtSND1/NST3 (Ohashi-Ito et al., 2010; Zhong et al., 2010c; Yamaguchi et al., 2011). These studies did not identify identical sets of genes, reflecting the different genes and different experimental strategies, but the sets showed some overlap. First, common targets of AtVND and AtSND1/NST3 include transcription factors such as MYB and ASYMMETRIC LEAVES2-LIKE/LATERAL ORGAN BOUNDARIES DOMAIN (ASL/LBD) (for details, please see sections below; Figure 2), and the genes encoding enzymes involved in SCW formation, such as the *IRX* genes (Taylor et al., 2003). Second, the VND proteins preferentially target genes involved in programmed cell death (nucleases, proteases, and metacaspases), and signal transduction (receptor-like kinases). Additionally, even for common targets, the transcriptional activation activities of AtVND and AtSND1/NST3 sometimes differ (Ohashi-Ito et al., 2010; Zhong et al., 2010c; Yamaguchi et al., 2011). *In silico* and *in vitro* analyses of *cis*-elements targeted by VNS revealed the 11-bp tracheary element-regulating *cis*-elements (TEREs; Pyo et al., 2007) and the 19-bp secondary wall NAC-binding elements (SNBEs; Zhong et al., 2010c), which partly overlap. Both *cis*-elements are enriched in promoter regions of the genes directly regulated by AtVND and AtSND1/NST3 (Pyo et al., 2007; Ohashi-Ito et al., 2010; Zhong et al., 2010c), and are recognized by both AtVND and AtSND1/NST3 in transient expression experiments (Zhong et al., 2010c), suggesting that the determination of target preference between the VND group and the NST group must be regulated by *cis*-elements other than TERE or SNBE.

The overall characteristics of VNS targets identified in Arabidopsis, including the *cis*-elements TERE and SNBE, are basically conserved in poplar, rice, maize, *B. distachyon*, and *Medicago truncatula* (Zhao et al., 2010; Zhong et al., 2010b, 2011; Ohtani et al., 2011; Valdivia et al., 2013). However, notably, the expression specificity depending on gene group that occurs in Arabidopsis was not detected in the other plant species. For example, in Arabidopsis stems, *AtVND* genes are preferentially expressed in xylem vessel cells, while *AtNST* genes are expressed in interfascicular fibers (Mitsuda et al., 2005, 2007; Zhong et al., 2006; Yamaguchi et al., 2008). By contrast, in poplar, rice, and maize, the *VNS* genes of both VND and NST groups are expressed in vessels and fibers (Zhong et al., 2010b, 2011; Ohtani et al., 2011). Thus, SCW formation in xylem vessels and fibers in those species must involve distinct layers of regulation for downstream events, in addition to transcriptional control. Work on the spatial organization of SCW in xylem vessel cells could inform our understanding of this. In the current model, the patterns of SCW deposition in xylem vessel cells can be regulated by the balance of assembly and disassembly of the cortical microtubule array, determining the spatial orientation of the CesA complex (Oda and Fukuda, 2013b). The interactions of specific proteins with microtubules and/or ROP-GTPase activities control this balance (Oda and Fukuda, 2012, 2013a). Certain SCW-related enzymes likely function in the apoplastic regions of plant cells (Schuetz et al., 2014), so we might have to think about the regulation of spatial activities of enzymes in each plant species.

### Transcriptional- and post-transcriptional regulation of *VNS* genes

Following the upregulation of *VNS* genes, the cells begin to differentiate as SCW-forming cells, such as xylem vessel cells and fibers. These cells are dead at maturity; therefore, *VNS* expression and/or activity must be well regulated in accordance with developmental programs and/or environmental signals.

During plant development, xylem vessel cells differentiate from vascular stem cells of the procambium and cambium (Fukuda, 1997; Demura and Fukuda, 2007). Phytohormones, especially auxin, provide one of first cues for vascular stem cell initiation. Several transcription factors function in the regulation of initiation, maintenance, and differentiation of vascular stem cells downstream of auxin signals (De Rybel et al., 2013; Ohashi-Ito et al., 2013a,b). Screening for factors upstream of AtVND7 in a transient expression system recently identified one such transcription factor, REV, a member of the Class III HD-Zip proteins (Carlsbecker et al., 2010; Miyashima et al., 2011; Endo et al., 2015). In addition, *LBD18/ASL20* and *LBD30/ASL19*, which are expressed in xylem vessels, can induce ectopic SCW deposition in various types of cells through the upregulation of *AtVND6* and *AtVND7* (Soyano et al., 2008). *LBD18/ASL20* and *LBD30/ASL19* are upregulated by auxin and by AtVND6 and AtVND7 (Soyano et al., 2008), and *LBD15/ASL11* and *LBD30/ASL19* are also direct targets of AtVND6 and/or AtVND7 (Ohashi-Ito et al., 2010; Zhong et al., 2010c; Yamaguchi et al., 2011). This indicates the existence of auxin-mediated feedback regulation between VND and LBD/ASL (Figure 2). Moreover, all *AtVND* genes can induce *AtVND7* expression by direct interaction with the *VND7* promoter region through regions containing the SMBE/TERE motif (Zhou et al., 2014; Endo et al., 2015). Thus, once the precursor cells initiate xylem vessel cell differentiation, the positive feedback transcriptional regulation efficiently upregulates VND activity. Transient expression assays identified GATA family members (GATA5 and GATA12), and other NAC proteins (ANAC075 and SND2) as upstream factors of *AtVND7* (Endo et al., 2015), suggesting that multiple signals contribute to the induction of VND activity through many types of transcription factors. Infection by the soil-born fungal pathogen *Verticillium longisporum* induces *AtVND7* expression, resulting in the transdifferentiation of vascular bundle cells into tracheary elements (Reusche et al., 2012). Recent work also showed that *AtVND6* and *AtVND7* expression increased in response to high salinity and iron depletion (Taylor-Teeples et al., 2015). Such abiotic and/or biotic stress signals might be mediated by specific types of transcription factors functioning upstream of *VNS* genes.

In addition to many positive regulators of *VNS* genes, as described above, other studies have identified negative regulators of VNS, including the WRKY-type transcription factor, WRKY12 (Wang et al., 2010). In the loss-of-function *wrky12* mutant, ectopic SCW formation occurred in pith parenchyma cells of inflorescence stems, and the expression of *NST2* increased. Recombinant WRKY12 protein can bind to the *NST2* promoter sequence *in vitro*; thus, WRKY12 negatively regulates SCW formation by directly inhibiting *NST2* expression in pith parenchyma cells (Wang et al., 2010). Additionally, protein-DNA interaction analysis in xylem-expressed transcription factors of Arabidopsis showed that E2Fc, a member of a transcription factor family conserved in eukaryotes and a negative regulator of endoreduplication in plants, may function as a key regulator of SCW formation (Taylor-Teeples et al., 2015). Notably, E2Fc seems to function as both an activator and a repressor of *AtVND6* and *AtVND7* expression, depending on the situation. E2Fc also can bind to the promoters of many kinds of xylem-expressed transcription factors in addition to the *AtVND* promoters (Taylor-Teeples et al., 2015). These complex interactions among transcription factors suggest that vascular plants have developed a robust transcriptional regulation system to promote xylem vessel cell differentiation, which is vital for land plants.

Post-transcriptional regulation also likely plays an important role in modulating VNS activity. A *VNS* gene in the poplar *P. trichocarpa*, *PtrWND1B/PtVNS11/PtrSND1-A2*, has alternative splicing variants that vary in abundance in different tissues (Li et al., 2012b; Zhao et al., 2014). The predicted protein product of the short and minor variant lacks the C-terminal region, but can bind to full-length PtVNS proteins. As a result, the truncated PtrWND1B/PtVNS11/PtrSND1-A2 inhibits transcriptional activation by PtVNS proteins (Li et al., 2012b), and can suppress the SCW thickening of fiber cells in poplar (Zhao et al., 2014). This alternative splicing regulation is completely dependent on the intron sequence of *PtrWND1B/PtVNS11/PtrSND1-A2*, and such regulation could be specific to poplar. Understanding the contributions of regulation of splicing to the control of VNS activity will require further survey of *VNS* genes.

NAC domain proteins form homo- and/or hetero-dimers (Olsen et al., 2005; Weiner et al., 2012). Indeed, yeast two-hybrid screens showed that AtVND and AtNST can bind each other to form hetero-dimers as well as forming homo-dimers (Yamaguchi et al., 2008; Li et al., 2012b). Transient expression assays on poplar *VNS* genes indicate that transactivation activity varies by PtVNS, even between “twin” genes that possess more than 90% similarity in amino acid sequence (Ohtani et al., 2011). Thus, the VNS hetero-dimers and homo-dimers should have different transactivation activities and we should consider the effects of dimerization, particularly when multiple *VNS* are expressed. Yeast two-hybrid screening for proteins that interact with VND7 also identified the NAC domain protein VND-INTERACTING2 (VNI2) as a key regulator of VND7 activity (Yamaguchi et al., 2010b). VNI2 acts solely as a transcriptional repressor and inhibits transcriptional activation activities of AtVND7 and xylem vessel cell differentiation, probably through direct interaction via the NAC domain regions (Yamaguchi et al., 2010b). A Recent work also reported ANAC103 as a possible interactor with AtVND7 in vascular tissues (Yamaguchi et al., in press). Taken together with the fact that proteasome-mediated proteolysis actively regulates AtVND7 (Yamaguchi et al., 2008), these findings show that multiple layers of regulation also affect AtVND7 at the protein level.

## SCW-related MYB proteins

### MYB transcription factors as lignin biosynthesis regulators

MYB transcription factors occur widely in eukaryotes and have characteristic, highly conserved DNA-binding domains, called the R1, R2, and R3 domains, at their N-termini. In plants, the majority of MYB proteins have only two domains and thus are called R2R3-MYB proteins; R2R3-MYB proteins are encoded by 126 genes in Arabidopsis (Stracke et al., 2001; Dubos et al., 2010), 109 in rice (Yanhui et al., 2006), 141 in eucalyptus (Soler et al., in press), and 192 in poplar (Wilkins et al., 2009). These R2R3-MYB family genes function in a wide range of developmental processes, stress responses, and metabolism (Jin and Martin, 1999; Stracke et al., 2001; Larkin et al., 2003; Grotewold, 2006; Lepiniec et al., 2006; Valliyodan and Nguyen, 2006; Bergmann and Sack, 2007; Chinnusamy et al., 2007; Ishida et al., 2008; Dubos et al., 2010; De Geyter et al., 2012; Grima-Pettenati et al., 2012; Muñoz-Nortes et al., 2014).

Studies in the 1990s revealed the involvement of MYBs in biosynthesis of phenylpropanoids. Since then, promoter analysis of phenylpropanoid biosynthetic genes, including *PAL* (encoding phenylalanine ammonia-lyase) and *4CL* (encoding 4-coumarate CoA ligase), revealed several important *cis*-elements in their promoters (Lois et al., 1989; Ohl et al., 1990; Becker-André et al., 1991; Leyva et al., 1992; Hauffe et al., 1993; Hatton et al., 1995; Wanner et al., 1995). One of them is the AC element, also known as the C1-motif, PAL-box, or H-box, which is rich in the sequence AC and is critical for xylem-specific expression of *PAL* and *4CL* (Leyva et al., 1992; Hauffe et al., 1993; Bell-Lelong et al., 1997). Bioinformatic and biochemical analyses showed that the AC elements share sequence similarity to the motif recognized by MYB transcription factors, and that, indeed, some MYBs bind to the AC elements to regulate gene expression (Romero et al., 1998). These findings led to genome-wide *in silico* analysis of presumed lignin biosynthesis genes of Arabidopsis, which found that almost all lignin biosynthesis genes have AC elements in their promoter regions (Weisshaar and Jenkins, 1998; Rogers and Campbell, 2004). Taken together, these findings suggest that *MYB* genes coordinately regulate lignin biosynthesis genes through the AC elements. Moreover, functional analysis of *MYB* genes expressed in secondary xylem of *P. taeda* (*PtMYB4*) and *E. gunnii* (*EgMYB2*) demonstrated that they can enhance lignin biosynthesis and/or SCW thickening when overexpressed (Patzlaff et al., 2003a; Goicoechea et al., 2005; Table 2). MYB proteins have been reported to be involved in lignin biosynthesis in many plant species (Grima-Pettenati et al., 2012); in this review we limit our focus to Arabidopsis *MYB* genes.

**Table 2:** ***MYB* genes related to SCW formation**.

**Gene Name**	**Locus or Transcript ID**	**Accession No**.	**Transcriptional activity**	**References**
***Arabidopsis thaliana***				
AtMYB4	At4g38620		Repressor	Jin et al., 2000
				Wang and Dixon, 2011
AtMYB6	At4g09460		Unknown	Zhong and Ye, 2012
AtMYB7	At2g16720		Repressor	Wang and Dixon, 2011
				Zhong and Ye, 2012
AtMYB20	At1g66230		Unknown	Zhong et al., 2008
				Nakano et al., 2010
AtMYB32	At4g34990		Repressor	Preston et al., 2004
				Wang and Dixon, 2011
AtMYB42	At4g12350		Unknown	Zhong et al., 2008
AtMYB43	At5g16600		Unknown	Zhong et al., 2008
				Nakano et al., 2010
AtMYB46	At5g12870		Activator	Ko et al., 2009
				Zhong et al., 2007a
				Nakano et al., 2010
AtMYB52	At1g17950		Activator/	Zhong et al., 2008
			Repressor	Nakano et al., 2010
				Cassan-Wang et al., 2013
AtMYB54	At1g73410		Activator	Zhong et al., 2008
AtMYB58	At1g16490		Activator	Zhou et al., 2009
AtMYB63	At1g79180		Activator	Zhou et al., 2009
				Nakano et al., 2010
AtMYB69	At4g33450		Activator	Zhong et al., 2008
AtMYB83	At3g08500		Activator	McCarthy et al., 2009
AtMYB85	At4g22680		Activator	Zhong et al., 2008
				Nakano et al., 2010
AtMYB99	At5g62320		Unknown	Nakano et al., 2010
AtMYB103	At1g63910		Activator	Zhong et al., 2008
				Nakano et al., 2010
				Öhman et al., 2013
***Eucalyptus gunnii***				
EgMYB2		AJ576023	Activator	Goicoechea et al., 2005
***Oryza sativa***				
OsMYB46	Os12g0515300/ LOC_Os12g33070	JN634084	Activator	Zhong et al., 2011
***Pinus taeda***				
PtMYB1		AY356372	Activator	Bomal et al., 2008
				Patzlaff et al., 2003b
PtMYB4		AY356371	Activator	Patzlaff et al., 2003a
PtMYB8		DQ399057	Activator	Bomal et al., 2008
***Populus trichocarpa***				
PtrMYB002	Potri.001G258700	KF148677	Activator	McCarthy et al., 2010
PtrMYB003	Potri.001G267300	KF148675	Activator	Wilkins et al., 2009
PtrMYB020	Potri.009G061500	KF148676	Activator	Zhong et al., 2013
PtrMYB021	Potri.009G053900	KF148678	Activator	
***Zea mays***				
ZmMYB46		JN634085	Activator	Zhong et al., 2011

### AtMYB46 and AtMYB83: second-layer master switches of SCW biosynthesis

**Figure 4:**
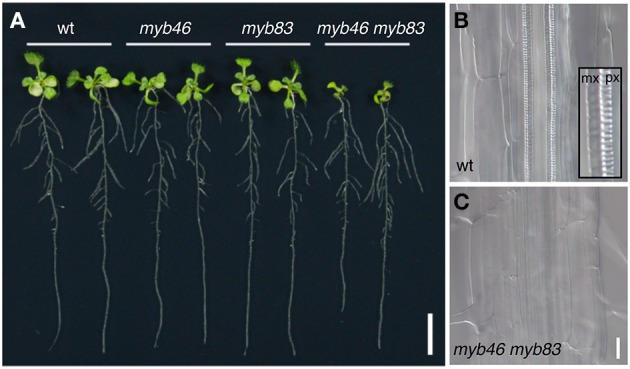
**The *MYB46/83* genes function as second-layer master switches for secondary cell wall formation. (A)** Ten-day-old Arabidopsis seedlings of wild type (wt), *myb46*, *myb83*, and *myb46 myb83* mutants. The *myb46 myb83* mutant shows growth inhibition in aerial parts. **(B,C)** Xylem vessels in the roots of the wild type (wt, **B**) and *myb46 myb83* mutants **(C)**. In the wild type, thick secondary cell wall is deposited in protoxylem-type (px) and metaxylem-type (mx) vessel cells (inset in **B**). In the *myb46 myb83* mutant, secondary cell wall deposition in xylem vessel cells is strongly inhibited, as described in McCarthy et al. (2009). Bars = 1 cm **(A)** and 25 μm **(B,C)**.

After the emergence of the first sets of evidence showing the involvement of pine and *Eucalyptus* MYBs in SCW formation in woody species (Patzlaff et al., 2003a,b; Goicoechea et al., 2005), their Arabidopsis closest functional orthologs MYB46 and MYB83 (*AtMYB46* and *AtMYB83*) were reported to function as key regulators of SCW formation (Zhong et al., 2007a; Ko et al., 2009; McCarthy et al., 2009; Table 2). These genes are preferentially expressed in xylem tissues, and their overexpression induced ectopic deposition of SCW. Conversely, expression of the chimeric repressors for AtMYB46 or AtMYB83 inhibited SCW deposition in xylem (Zhong et al., 2007a; McCarthy et al., 2009). Promoter activity of *AtMYB46* was found in both protoxylem-type and metaxylem-type vessel cells, suggesting the involvement of these MYBs in xylem vessel cell formation (Nakano et al., 2010). Consistent with this observation, in the double mutant *myb46 myb83*, SCW deposition in vessel cells is severely affected, leading to seedling growth arrest in the mutant (McCarthy et al., 2009; Figure 4). These findings indicate that AtMYB46 and AtMYB83 redundantly regulate SCW formation in Arabidopsis (McCarthy et al., 2009). Importantly, AtVND and/or AtNST/SND, the master regulators of the differentiation of SCW-containing cells, directly target these *MYB* genes (Zhong et al., 2007a, 2010c; McCarthy et al., 2009; Ohashi-Ito et al., 2010; Yamaguchi et al., 2011). Thus, AtMYB46 and AtMYB83 act as second layer-master switches of SCW biosynthesis (Figure 2).

### Downstream genes of second-layer master switch MYBs

#### Biosynthetic genes of SCW components

The regulatory target genes of AtMYB46 include many downstream factors involved in SCW formation (Zhong et al., 2007a; Zhong and Ye, 2012; Ko et al., 2009, 2012; Kim et al., 2012, 2013a,b, 2014a,b). Overexpression analysis of *AtMYB46* and time-course transcriptome analysis using an AtMYB46-dependent SCW formation induction system revealed that AtMYB46 can upregulate a number of SCW-biosynthesis genes (Zhong et al., 2007a; Zhong and Ye, 2012; Kim et al., 2013a,b, 2014a,b). Recently, independent groups defined the *cis*-element sequence recognized by MYB46 and/or MYB83 as the secondary wall MYB-responsive element [SMRE; ACC(A/T)A(A/C)(T/C), Zhong and Ye, 2012] or MYB46-responsive *cis*-regulatory element [M46RE; (T/C)ACC(A/T)A(A/C)(T/C), Kim et al., 2012], both of which contain the AC element sequence originally identified in the *PAL* gene promoter (Lois et al., 1989; Ohl et al., 1990; Leyva et al., 1992; Hatton et al., 1995; Wanner et al., 1995). A genomic survey of these *cis*-elements and DNA-protein binding assays suggest that the direct targets of AtMYB46/83 include genes encoding transcription factors (*KNAT7*, *MYBs*, and *AtC3H14*), the suite of SCW biosynthetic genes, including SCW-specific cellulose synthase genes (*CesA4*, *CesA7*, and *CesA8*), xylan biosynthetic genes (*IRX7/FRA8*, *IRX8*, *IRX9*, and *IRX14*), a mannan synthesis gene (*CSLA9*), and lignin biosynthetic genes (*PAL1*, *C4H*, *4CL1*, *C3H1*, *HCT*, *CCoAOMT*, *CCR1*, *F5H1*, *CAD6*, and laccases), and genes related to cytoskeleton regulation and signal transduction (Zhong and Ye, 2012; Kim et al., 2013a,b, 2014a,b,c). Of note, some AtMYB46/83 direct targets overlap with the direct targets of VNS proteins, such as *CesA4* and *CesA8* (Ohashi-Ito et al., 2010; Zhong et al., 2010c; Yamaguchi et al., 2011). *In vitro* DNA-protein binding assays revealed that such “feed-forward” regulation often occurs in the transcriptional regulatory network in xylem cells (Taylor-Teeples et al., 2015). However, in the case of Arabidopsis, AtMYB46/83 appears to have a greater contribution than VNS, at least for cellulose synthase gene activation, as demonstrated by the severe defects in SCW deposition in xylem vessels of the *myb46 myb83* double mutant (McCarthy et al., 2009; Figure 4) and the failed complementation of the *cesa* mutant phenotype by M46RE-mutated promoter-driven *CesA* genes (Kim et al., 2013a). Further study on the mechanisms by which VNS and MYB46/83 generate SCW will fill the gap between the *in vitro* DNA-protein interactions and *in vivo* mutant phenotypes.

#### MYB transcription factors downstream of AtMYB46/83; intermediate regulators of SCW formation

In addition to the genes for biosynthesis of SCW components, transcriptome analysis identified more than 40 transcription factors downstream of AtMYB46/83 (Ko et al., 2009; Kim et al., 2012; Zhong and Ye, 2012). This list includes KNAT7, NACs, MYBs, and AtC3H14, some of which function in SCW formation (Zhong et al., 2008; Ko et al., 2009; Zhou et al., 2009; Li et al., 2011, 2012a; Wang and Dixon, 2011; Kim et al., 2012; Zhong and Ye, 2012; Cassan-Wang et al., 2013; Öhman et al., 2013). Here we focus on AtMYB transcription factors downstream of AtMYB46/83 and describe the other factors in subsequent sections.

Before their identification as direct targets of AtMYB46/83, several AtMYBs had been reported to function in lignin biosynthesis. AtMYB4, an active repressor, regulates the expression of *C4H*, which encodes a cinnamic acid 4-hydroxylase required for biosynthesis of all types of lignin monomers, and AtMYB32 and AtMYB7, close homologs of AtMYB4, also negatively regulate several lignin biosynthesis genes (Jin et al., 2000; Preston et al., 2004). These repressor MYBs downregulate the expression of *AtNST3/SND1 in vitro*, and AtNST3/SND1 directly regulates *AtMYB32* (Wang and Dixon, 2011). Based on this observation, negative feedback regulation of the VNS-MYB network for fine-tuning of SCW formation has been suggested (Wang and Dixon, 2011; Figure 2). Moreover, the lignin-specific MYBs, AtMYB58, AtMYB63, and AtMYB85, regulate lignin biosynthesis, but not cellulose and hemicellulose deposition, because their overexpression induced ectopic deposition of only lignin (Zhong et al., 2008; Zhou et al., 2009). These genes are expressed in lignifying cells such as xylem vessels and fibers, and are probably regulated by both AtVNS and AtMYB46/83 (Zhong et al., 2006, 2007a,b; Ko et al., 2009; Kim et al., 2012; Zhong and Ye, 2012). AtMYB58 and AtMYB63 can bind the AC element, and AtMYB85 can activate the promoter activity of *4CL*; thus they can activate the monolignol biosynthesis pathway (Zhong et al., 2008; Zhou et al., 2009). Recent work on AtMYB103 revealed that the *myb103* mutant shows a strong reduction of syringyl lignin, possibly due to a decrease in *F5H* expression (Öhman et al., 2013). These results suggest that the primary function of AtMYB103 is in regulation of lignin biosynthesis and that AtMYB103 functions as one of the lignin-specific MYBs, although *in vitro* transient expression assays showed the AtMYB103 can upregulate *CesA8* promoter activity (Zhong et al., 2008).

The last group of MYB46/83-downstream MYBs contains AtMYB42, AtMYB43, AtMYB52, and AtMYB54, which are preferentially expressed in xylem tissues (Zhong et al., 2008; Nakano et al., 2010). However, their functions in SCW formation remain controversial. Overexpression of dominant-repressor forms of MYB52 and MYB54 inhibit SCW deposition in interfascicular fibers and vessels, but overexpression of *MYB52* and *MYB54* produced no significant changes (Zhong et al., 2008). Recently, AtMYB52 was suggested to negatively regulate SCW formation, because the *myb52* mutant showed ectopic lignin deposition and the expression of *AtMYB52* and SCW-related genes showed a high degree of correlation (Cassan-Wang et al., 2013). AtMYB46 and AtMYB83 can upregulate *AtMYB43* (Nakano et al., 2010), but a detailed functional analysis remains to be performed.

#### Other MYBs that function in SCW formation

Several additional MYB transcription factors also participate in the regulation of SCW biosynthesis, probably in an MYB46/83-independent manner. Zhong et al. demonstrated that *AtMYB20* and *AtMYB69* also function downstream of AtNST1 and/or AtNST3/SND1, and are preferentially expressed in xylem cells (Zhong et al., 2006, 2007a,b, 2008). Dominant-repression analysis showed AtMYB69 is involved in the regulation of SCW formation (Zhong et al., 2008), and *AtMYB69* is among the top 60 genes co-expressed with *AtMYB52* (Cassan-Wang et al., 2013). In addition, *AtMYB75* has been shown to be involved in SCW formation of xylem tissues (Bhargava et al., 2010). *AtMYB75* is also called *PRODUCTION OF ANTHOCYANIN PIGMENT1 (PAP1)*, because this gene was earlier identified as a positive regulator of anthocyanin biosynthesis (Borevitz et al., 2000). Phenotypes of loss-of-function mutant and overexpressor of *AtMYB75* suggested that AtMYB75 negatively regulates SCW biosynthesis, especially for the branch of phenylpropanoid pathway connected to lignin biosynthesis (Bhargava et al., 2010). Nakano et al. (2010) identified *AtMYB99* in a survey of factors upregulated during *in vitro* differentiation of xylem vessel cells. *AtMYB99* is expressed in xylem vessel cells from early stages, suggesting its involvement in SCW formation in vessels (Nakano et al., 2010). However, the contribution of these genes to SCW formation in xylem tissues remains unknown. Further experiments will give us clues to elucidate the function of these MYBs.

In addition to the MYBs described above, AtMYB26 has been known to regulate SCW formation in anther endothecium (Wilson et al., 2011). Arabidopsis *myb26* mutant, also known as *male sterile35* mutant, lacks SCW in anther endothecium, resulting in a failure of anther dehiscence and male sterility (Dawson et al., 1999; Steiner-Lange et al., 2003). Similar phenotypes are found in *nst1 nst2* double mutant (Mitsuda et al., 2005), and overexpression of *AtMYB26* induced ectopic deposition of SCW (Yang et al., 2007) as the cases of *AtNST1* and *AtNST2* overexpression (Mitsuda et al., 2005). Interestingly, *AtNST1* overexpression can induce *AtMYB26* expression (Mitsuda et al., 2005), and adversely *AtMYB26* overexpression can upregulate *AtNST1* and *AtNST2* expressions (Yang et al., 2007). AtMYB26 shares relatively high sequence homology with AtMYB46/83, second-layer master switches of SCW biosynthesis in xylem (Zhao and Bartley, 2014). Therefore, these findings seem to suggest that in the case of anther endothecium, the relationship between NAC and MYB had been changed to make a positive transcriptional feedback loop rather than the transcriptional regulation cascade, probably to make it possible to complete SCW biosynthesis in a short time of anther stage 11, which can be estimated up to 48 h (Smyth et al., 1990; Sanders et al., 1999), during another development.

#### Evolutionary conservation of MYB46/83 function as second-layer master switches for SCW formation

The data described above indicate a complex network of MYB-mediated transcriptional regulation of SCW formation (Figure 2). The VNS proteins act as the primary master switches of woody cell differentiation, and the MYB46/83 proteins act as secondary master switches of SCW formation. Downstream of the MYB46/83, several groups of MYBs mediate the transcriptional signals that regulate SCW biosynthetic processes; some MYBs specifically control lignin biosynthesis, and some MYBs repress or enhance SCW biosynthesis, at least partially (Figure 2). Signals can pass to the master switches from these MYBs (Wang and Dixon, 2011; Figure 2). SCW features, such as composition of cellulose, hemicellulose, and lignin, and S/G ratio of lignin, vary with tissue type, plant age, plant species, and environmental stress (Campbell and Sederoff, 1996; Knox, 2008; Vogel, 2008; Pauly and Keegstra, 2010). Complex networks of MYBs likely operate as a fine-tuning system for the formation of SCW with the appropriate composition for the specific situation.

Work to date has identified several orthologs and putative functional homologs of *AtMYB46* from vascular plants, including poplar, pine, spruce, rice, maize, and switchgrass (*Panicum virgatum*), in addition to *PtrMYB4* and *EgMYB2* (Patzlaff et al., 2003a,b; Goicoechea et al., 2005; Bedon et al., 2007; Bomal et al., 2008; Zhong et al., 2011, 2013; Zhao and Bartley, 2014). Commonly, these MYBs can bind to the AC-rich elements, and overexpression of these MYBs upregulates lignin deposition (Patzlaff et al., 2003a,b; Goicoechea et al., 2005; Bomal et al., 2008; Zhong et al., 2011, 2013). These facts strongly suggest evolutionary conservation of second-layer master switch MYBs for SCW formation among vascular plant species. However, given the different characteristics of the SCW in different plant species and the fact that MYBs represent one of the most-expanded gene families in plants, the numbers and functions of intermediate MYBs downstream of MYB46/83 may have diversified among vascular plants. Engineering of lignin modification is an important target for industrial uses of plant materials (Pauly and Keegstra, 2010; Simmons et al., 2010; Zeng et al., 2014); thus we anticipate further studies of MYBs in crop species, particularly in woody species and biofuels feedstocks.

## Other transcription factors involved in SCW formation

Finally, we would like to mention additional important transcription factors involved in SCW formation. Two NAC transcription factors, AtSND2 and AtSND3, which are expressed in SCW-associated tissues, function downstream of VNS proteins for SCW formation (Zhong et al., 2008). Hussey and his co-workers showed that AtSND2 can influence almost all the regulatory programs involved in SCW formation, i.e., biosynthesis of cellulose and hemicellulose, lignin polymerization, and signal transduction, in addition to the expression of *AtNST3/SND1* (Hussey et al., 2011). Overexpression of *AtSND2* in *Eucalyptus* increased the thickness of the SCW in fiber cells; thus the molecular function of SND2 is basically conserved between herbaceous and woody plants (Hussey et al., 2011). However, overexpression phenotypes differ in the woody species and transgenic lines; the effects of *AtSND2* overexpression in Arabidopsis differed between Zhong et al. (2008) and Hussey et al. (2011), and the *AtSND2* overexpression in *Eucalyptus* showed the increase in SCW thickness, while overexpression of *PopNAC154*, one of poplar genes homologous to *AtSND2*, did not change SCW thickness in xylem tissues of poplar (Grant et al., 2010; Hussey et al., 2011). These observations suggest that AtSND2 and its orthologs function as key modulators of SCW formation, and the effects of overexpression may change depending on the situation.

Transcriptome analysis of an *AtMYB46*-overexpressing line identified the plant-specific tandem CCCH zinc-finger gene *AtC3H14* as a direct target of AtNST3/SND1 and AtMYB46 (Ko et al., 2009). AtC3H14 can activate transcription of the genes for cellulose, hemicellulose, and lignin biosynthesis (Ko et al., 2009; Kim et al., 2014b). Also, a recent study proposed an additional role of AtC3H14 in post-transcriptional regulation (Kim et al., 2014b). AtC3H14 can directly bind to mRNA in a target sequence-specific manner, similar to animal tandem CCCH zinc-finger proteins (Blackshear, 2002), and some cell wall-related genes seem to be binding targets of AtC3H14. Thus, AtC3H14 might participate in post-transcriptional and transcriptional regulation of cell wall biosynthetic genes (Kim et al., 2014b). Further functional analysis of AtC3H14 might reveal a new regulatory layer in the current model of SCW formation by transcription factors.

Moreover, other types of transcription factors, namely the homeodomain protein KNOTTED ARABIDOPSIS THALIANA7 (KNAT7) and OVATE FAMILY PROTEIN 4 (OFP4), have been described as negative regulators of SCW biosynthesis. Arabidopsis *KNAT7* was first identified by co-expression analysis with SCW-related enzyme genes (Brown et al., 2005; Ehlting et al., 2005; Persson et al., 2005). The *knat7* mutant showed a xylem phenotype similar to *irx* mutants; thus KNAT7 was also named IRX11 (Brown et al., 2005). AtNST3/SND1 and AtMYB46 directly target *KNAT7* (Zhong et al., 2008; Ko et al., 2009). Li et al. revealed that KNAT7 functions as a transcriptional repressor, and that OFP4 can enhance KNAT7 activity via physical interaction. Both *KANT7* and *OFP4* are expressed in SCW-forming xylem cells (Li et al., 2011, 2012a), but, interestingly, the effects of *knat7* loss-of-function mutation differ in xylem vessels and fiber cells. In xylem cells the SCW thickness decreased, leading to the *irx* phenotype (Brown et al., 2005; Li et al., 2012a). By contrast, the SCW thickness increased in fiber cells (Li et al., 2012a). Based on the identification of several interaction partners of KNAT7, including OFP4, MYB75/PAP1, and BELL1-LIKE HOMEODOMAIN6 proteins (Hackbusch et al., 2005; Bhargava et al., 2010; Li et al., 2011; Liu et al., 2014), KNAT7 may regulate specific aspects of SCW formation, depending on cell type, through interaction with different partners (Li et al., 2012a; Liu et al., 2014). The molecular function of KNAT7 is conserved with its poplar ortholog (Li et al., 2012a), and KNAT7 appears to have developed as a negative regulator to fine-tune SCW biosynthesis to fit the situation.

## Conclusion and perspectives

During the last decade, extensive research has produced remarkable progress in our understanding of transcriptional regulation of SCW formation. Although some key modulators may remain unknown, the current model (Figure 2) covers the essential players of SCW formation regulation. SCW in wood tissues provides a major source of land biomass, and SCW formation thus is an important target for biomass engineering. Several trials using the transcription factors described in this review to target SCW properties have been already reported (Carpita, 2012; Yang et al., 2013; Sakamoto and Mitsuda, 2015). Such applied research will assume growing importance in studies of the regulation of SCW formation.

In 2015, Taylor-Teeples et al. reported a protein–DNA interaction-based network between transcription factors and SCW metabolic genes of Arabidopsis (Taylor-Teeples et al., 2015). This map showed complex interactions among transcription factors and SCW metabolic genes, with many instances of feed-forward regulation (Figure 2A). In the protein–DNA interaction network, many kinds of transcription factors other than those previously reported could recognize the promoter sequence of transcription factors and SCW metabolic genes (Taylor-Teeples et al., 2015). This observation implies that we are now at a turning point in research on the transcriptional regulation of SCW biosynthesis: in addition to continuing our efforts to identify genes involved in SCW formation and reveal the interactions between them on a one-on-one basis, we must move to the next steps. Thus, future research must aim to reveal the dynamism of the network itself based on observations of what happens *in vivo*, because modeling based on *in vitro* data only tells us the many possibilities of the network. The transcriptional regulatory network of SCW formation could become a good model for such advanced analysis in plants.

**Figure 5:**
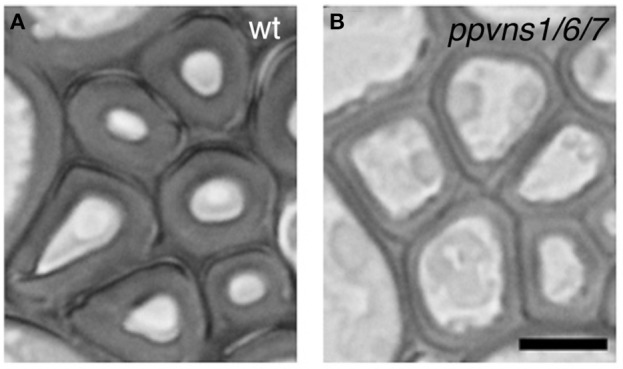
**The *PpVNS* genes function in cell wall thickening in the moss *P. patens***. Stereid cells in leaf vein of the wild type **(A)** and *ppvns1 ppvns6 pvns7* triple mutant **(B)**. In the triple mutants, the stereid cell walls were significantly less thick, suggesting the importance of PpVNS proteins in cell wall thickening in the moss. Data were adapted from Xu et al. (2014). Bar = 5 μm.

Comparative transcriptomic work revealed that the xylem transcriptomes of vascular plants are more highly conserved than the overall transcriptomes (Li et al., 2010), indicating the ancient origin of the xylem transcriptome. In accordance with this idea, recent work showed that the scheme of VNS-based transcriptional regulation of cell differentiation for wall thickening is conserved in the moss *Physcomitrella patens* (Figure 5), although *P. patens* does not have vascular plant-type SCW (Xu et al., 2014). The *P. patens* genome has eight *VNS* loci (Figure 3A), and the triple mutant *ppvns1 ppvns6 ppvns7* of *P. patens* showed reduced wall thickness in stereid cells, which serve as supporting cells in mosses (Figure 5). Transcriptome analysis of *P. patens* overexpressing *PpVNS7* indicated that PpVNS regulates many putative orthologs of the direct targets of AtVNS, including putative orthologs of AtMYB46/83/103 and AtMYB85 (Xu et al., 2014). These findings suggest that the genes downstream of VNS are evolutionarily conserved, and that the VNS-MYB-based transcriptional regulatory system of wall modification has an ancient root, at least at the common ancestors of mosses and vascular plants. Land plants would have developed this core regulatory scheme of cell wall modification during evolution to adapt to new environments. As reviewed here, we now have extensive knowledge on the factors governing SCW formation in a wide range of plant species, which probably includes some species-specific elements. What is the particular regulatory scheme for each species? Which parts of the common regulatory module are conserved among land plants? Future work will provide clues to answer these questions.

### Conflict of interest statement

The authors declare that the research was conducted in the absence of any commercial or financial relationships that could be construed as a potential conflict of interest.
